# The novel coronavirus (COVID-19) and unintended pregnancy during the quarantine period

**DOI:** 10.11604/pamj.supp.2020.35.2.23313

**Published:** 2020-05-11

**Authors:** Mansoureh Yazdkhasti

**Affiliations:** 1 Social Determinants of Health Research Center, Department of Midwifery, school of medicine, Alborz University of Medical Sciences, Karaj, Iran

**Keywords:** COVID-19, public health, unwanted pregnancy

## To the editors of the Pan African Medical Journal

The novel coronavirus (COVID-19) is highly similar to the Severe Acute Respiratory Syndrome (SARS) and is responsible for the 2019-2020 pandemic. The World Health Organization (WHO) has considered the vast prevalence of COVID-19 as a public health emergency [1]. Quarantine raises an important need for interventions to reduce unintended pregnancy. In the quarantine period, traffic restrictions imposed by governments result in reduces access to family planning equipment/contraceptives. With regard to reproductive health, the number of unintended pregnancies is expected to increase during the quarantine period resulting from the global encounter with COVID-19. Unintended pregnancy as a key challenge for the health sector imposes considerable socioeconomic costs on the society. In other words, from the socioeconomic perspective, unwanted pregnancy affects reproductive indices in the health system, thereby decreasing the quality of life and work force productivity [2].

In addition to unintended pregnancy, COVID-19 as a highly infectious disease can lead to high-risk pregnancies. Studies have indicated that suffering from SARS during pregnancy resulted in spontaneous miscarriage, preterm labor pain (PLP), Intrauterine Growth Restriction (IUGR), endotracheal intubation, admission to intensive care units, and Disseminated Intravascular Coagulopathy (DIC) [3]. COVID-19 also seems to have similar complications to those of other infectious diseases although no sufficient evidence is available in this regard. Chen et al. conducted a study on pregnant women with COVID-19 in Zhongnan Hospital, Wuhan, China from 20 to 31 January 2020 and reported none of the above mentioned complications. Therefore, there is no evidence regarding the impact of COVID-19 on pregnancy [4]. Nonetheless, such an impact may be possible since infections can lead to early uterine contractions and eventually preterm delivery [5].

Unintended pregnancies have an adverse effect on a child’s health. Various micro-level investigations have revealed the effect of children´s health on their ability to learn and acquire knowledge. In addition, adults´ health status could affect the labor force volume, absence from work, and workers´ efficiency. Moreover, macro-level studies have indicated that population´s health had a considerable impact on each country´s economic growth rate [6]. Unintended pregnancy may be accompanied with the probability of abortion. Considering the quarantine period, the number of unsafe abortions may increase particularly in developing countries. Unsafe abortions can lead to negative mental consequences, disability, and even maternal mortality. These can damage human work force quality and impose great expenditures on the health system that are not even recorded in the health sector´s balance sheets [7]. During the pandemic, most costs of the health sector taxpayers may also be allocated to unintended pregnancies and their reproductive outcomes.

## Conclusion

Overall, unintended pregnancy and its undesirable maternal-infantile outcomes will increase the short- and long-term economic and psychosocial expenditures of this global crisis. As mentioned above, the COVID-19 pandemic may increase the probability of unintended pregnancy. Considering the limited access to contraceptives, particularly in poor and developing countries, during the quarantine period, they are recommended to be provided to families for free. In other words, contraceptives are suggested to be added to the families´ baskets of goods. Families and the youth are also recommended to be provided with online or offline educational applications on reproductive and sexual health for free. Prevention and control of unintendedpregnancy can, in turn, help save the government´s general budget and decline the healthcare cost inflation.

## Competing interests

The author declares no competing interests.
